# Efficacy of Adjunctive Treatments Added to Olanzapine or Clozapine for Weight Control in Patients with Schizophrenia: A Systematic Review and Meta-Analysis

**DOI:** 10.1155/2015/970730

**Published:** 2015-01-13

**Authors:** Yun-Jung Choi

**Affiliations:** Red Cross College of Nursing, Chung-Ang University, 84 Heukseok-Dong, Dongjak-Gu, Seoul 156-756, Republic of Korea

## Abstract

*Objectives*. This study was conducted to review systematically adjunctive treatments for weight reduction in patients with schizophrenia and compare efficacies of clinical trials through meta-analysis, so as to provide effective clinical guideline regarding weight control for patients taking atypical antipsychotics. *Methods*. Candidate clinical trials were identified through searching the Cochrane Central Register of Controlled Trials, PubMed, and PsycINFO. Fourteen randomized clinical trials were included for systematic review and meta-analysis from 132 potential trials. The Comprehensive Meta-Analysis version 2 was used for meta-analysis. *Results*. Difference in means and significances from meta-analyses regarding weight control by adjunctive treatments showed that topiramate, aripiprazole, or sibutramine was more effective than metformin or reboxetine. Psychiatric evaluations did not show statistically significant changes between treatment groups and placebo groups except topiramate adjunctive treatments. Adverse effects regarding adjunctive therapies were tolerable and showed statistically no significances compared to control groups. *Conclusion*. Though having several reports related to exacerbation of psychiatric symptoms, topiramate and aripiprazole are more efficacious than other medications in regard to weight reduction and less burden of critical adverse effects as well as being beneficial for clinical improvement.

## 1. Introduction

Patients with schizophrenia are predisposed to becoming overweight through lifestyle factors, including sedentary lives, unhealthy diet, and socioeconomic status [1]. Though research into underlying mechanisms has identified some risk factors such as H1 receptor affinity [2] and 5HT2c polymorphisms [3], the pharmacology of antipsychotic-induced weight gain is largely not understood and is very likely multifactorial [4]. In schizophrenia, the estimated prevalence of overweight and obese individuals is 2- to 3-fold that of the general population [5]. Prevention of weight gain and treatment of obesity among patients with schizophrenia taking atypical antipsychotics have become a priority in clinical practice and represent a major public health problem [1, 6]. While switching to a more weight-neutral atypical antipsychotic agent offers promise in halting or reversing weight gain associated with an antipsychotic agent, many patients and their clinicians are reluctant to risk worsening or return of psychotic symptoms [4]. As a result, various agents have been proposed as adjunctive treatments to attenuate antipsychotic-induced weight gain [7].

This study was conducted to review systematically adjunctive treatments for weight reduction in patients with schizophrenia and compare efficacies of clinical trials through meta-analysis, so as to provide effective clinical guideline regarding weight control for patients taking atypical antipsychotics.

## 2. Methods

### 2.1. Identification of Clinical Trials

**Figure 1:**
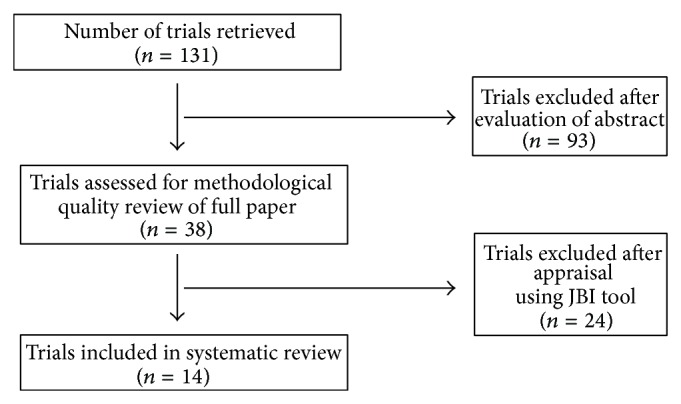
The trial selection process for inclusion.

Candidate clinical trials were identified through searching the Cochrane Central Register of Controlled Trials, PubMed, and PsycINFO. Relevant trials were searched using the following keywords: “weight gain,” “weight loss,” “weight reduction,” “antipsychotics,” “atypical,” “schizophrenia,” “adjunctive,” “additional,” “combine,” “coadministration,” “treatment,” “therapy,” “effect,” “efficacy,” and “clinical trial.” Reference lists of retrieved articles were searched for additional studies. Inclusion criteria for this research were treatment effectiveness from clinical trials of adjunctive treatments added on atypical antipsychotics for adult schizophrenia population and containing outcome data regarding weight and clinical evaluation. Primary outcomes included the reduction in weight determined by body weight (BWT) or body mass index (BMI). Secondary outcomes included psychiatric symptom evaluations measured by the brief psychiatric rating scale (BPRS), the positive and negative syndrome scale (PANSS), or the scale for assessment of positive symptom (SAPS). The exclusion criteria included research from nonpharmacological treatments, secondary data sources, unclear research procedures or outcomes, subjects with comorbid disorders, and population of children or adolescents. Initially, one hundred and thirty-one trials were retrieved by searching for keywords from the databases. Ninety-three trials were excluded after evaluation of abstracts. Full papers of thirty-eight potential trials for inclusion were reviewed using the Joanna Briggs Institute data extraction form [5] and twenty-four trials were excluded. The Joanna Briggs Institute data extraction form was used to extract data while reviewing full text paper, which contained author, source, method, sample size, and interventions of the study. Remaining fourteen trials were assessed by the Jadad scale for methodological quality. The Jadad scale was applied to assess quality of clinical trials. The Jadad scale is comprised of the following five questions: (1) Is the study randomized? (2) Is the study double blinded? (3) Is there a description of withdrawals? (4) Is the randomization adequately described? (5) Is the blindness adequately described? Each question demands a yes or no response. A total of five points can be awarded, and higher scores indicate superior quality [6]. A flow chart of the trial inclusion procedure is provided in Figure 1.

### 2.2. Data Extraction and Collation

Primary outcomes included the Jadad score, atypical antipsychotics as concurrent therapy, type and daily maximum dose of adjunction, treatment duration, sample size and number of drop, weight reduction and significance between groups as primary outcome, and psychiatric symptom evaluation and significance between groups as secondary outcome. Efficacies of adjunctive treatments were compared by primary as well as secondary outcomes including mean change from baseline. Meta-analyses were conducted using the Comprehensive Meta-Analysis version 2 regarding adjunctive treatments with topiramate, sibutramine, metformin, and reboxetine.

## 3. Results

Fourteen randomized clinical trials published from 2002 to 2010 were included for this systematic review and meta-analysis (Table 1). Mean of the Jadad scores was 3.8 out of 5 (ranged from 5 to 2). Medications for adjunctive treatments were aripiprazole, topiramate, metformin, sibutramine, reboxetine, famotidine, nizatidine, and fluoxetine. Adjunctive treatments of famotidine, nizatidine, and fluoxetine exhibited neither reductions of weight nor significances compared to the placebo groups (*P* = 0.91~0.36). Rest of the adjunctive treatments showed weight reductions from −6.8 to +5.5 kg (*P* = 0.31~<0.001). Difference in means and significances from meta-analyses regarding adjunctive treatments were as follows: topiramate (*n* = 99): −2.405 kg/m^2^ (*P* = 0.004); sibutramine (*n* = 58): −2.342 kg (*P* = 0.004); metformin (*n* = 160): −1.331 kg (*P* = 0.014); reboxetine (*n* = 85): −1.862 kg (*P* = 0.001) (Tables 2–5).

Psychiatric evaluations, evaluated by PANSS, BPRS, and SAPS, did not show statistically significant changes between treatment groups and placebo groups except topiramate adjunction (*P* ≤ 0.001). Adjunctive treatments of metformin plus sibutramine on olanzapine brought exacerbation of psychiatric symptoms evaluated by BPRS raised up to 35.4 mean scores from the baseline. Adverse effects regarding adjunctive therapies were tolerable and showed statistically no significances compared to control groups. Three participants among the fourteen trials experienced severe adverse effects, in aripiprazole adjunctive treatments, such as moderate sinus tachycardia, severe psychotic disorder, and severe auditory hallucinations.

## 4. Discussion

Since the occurrence of dilemma of weight gain in patients receiving atypical antipsychotics, various efforts have been tried to solve the problem. Present study shows the trends of trials by time transition. In the early 2000s, fluoxetine, nizatidine, and famotidine were examined as choices of adjunctive treatments for weight control regarding atypical antipsychotics [17, 18, 20]. However, those combinations have failed to present consistent weight reduction neither within the treatment group nor difference between the groups (*P* = 0.36~0.91) [17, 18, 20]. After the middle of the 2000s, reboxetine, sibutramine, and metformin have been investigated and there have been some degree of rewarding findings. Recently, topiramate and aripiprazole are added in the stream of finding a key of weight reduction for patients taking atypical antipsychotics [6, 9, 10].

Reboxetine is a selective norepinephrine reuptake inhibitor (NRI) and is a new psychotropic drug broadly used as an antidepressant and antianxiety agent, which has been reported to have effects on weight loss [21]. Lu et al. reported a case significant weight loss treated with reboxetine at 12 mg daily for a total duration of 11 months [22]. Through stimulation of NE activity by the selective NRI, reboxetine is considered to diminish olanzapine induced weight gain [19]. In present study, two clinical trials were examined, and the results were in-line with previous researches at a glance in which reboxetine showed less weight gain than placebo (*z* = −3.310, *P* = 0.001). However, the mean weight changes of the reboxetine groups were increased from the baseline (ranged from +2.4 kg to +3.3 kg). That means patients could get obese in spite of taking reboxetine for weight reduction, which reflects that the medication seems ineffective treatment option for weight reduction of patients prescribed atypical antipsychotics [23]. Reboxetine is known as a mediator of enhancing cognitive dysfunction in schizophrenia patients; long-term studies using higher dosages are needed to determine the role of reboxetine as cognitive enhancers in patients with schizophrenia and other disorders associated with cognitive impairments [22, 23]. Conversely, in this review, the reboxetine group did not show statistical difference of psychiatric symptom improvement compared to the placebo group. Though adverse effects were tolerable, seven out of eighty-five participants were withdrawn from the trials due to lack of efficacy. More evidence of its safety profile is warranted before promotions become widely accepted [22].

Sibutramine is a weight loss agent affecting both serotonin and norepinephrine reuptake, which partly mediates activation of the serotonin 5-HT2c receptor that associated with weight loss [7]. In a 16-week double-blind trial, the addition of sibutramine to an ongoing antipsychotic regimen was shown to have no significant differences between groups on mean loss of weight [24]. In this study, sibutramine adjunctive treatments were significantly effective on weight reduction compared to placebo (*z* = −2.877, *P* = 0.004). In the research by Henderson et al., the weight reduction in the sibutramine group has shown obvious effects compared to the placebo group (mean change from baseline, −6.8 kg versus +3.9 kg) [7]. Though overall adverse effects were tolerable and no subjects were withdrawn from present review for adverse events, systolic blood pressure was increased significantly in the sibutramine group, and one subject in the sibutramine group died during week 10 in this review by undetected coronary artery disease, despite a normal cardiac stress test within a year prior to the study [7].

Since 2002, several cardiovascular adverse events (hypertension, tachycardia, arrhythmias, and myocardial infarction) were reported in sibutramine-treated patients [7, 14, 24]. The Sibutramine Cardiovascular Outcomes Trial (SCOUT) confirmed that subjects with preexisting cardiovascular disease (CVD) on long-term treatment with sibutramine had a significantly increased risk for nonfatal myocardial infarction and nonfatal stroke but not cardiovascular death or all-cause mortality [25]. The US Food and Drug Administration (FDA) first stated that the drug should carry a “black box” warning because of an increased risk of stroke and heart attack in patients with a history of CVD. In October 2010, sibutramine was withdrawn from the US market [22].

Metformin is a hepatic-selective insulin sensitizer, which reduces weight, blood glucose, insulin, and hemoglobin A1c (HbA1c) levels in obese nondiabetic adults [12]. It has been demonstrated to improve glycaemic control and promotes a moderate weight loss in both diabetic and nondiabetic subjects [11, 13]. Metformin is particularly attractive because of its dual mechanism of decreasing body weight gain and improving insulin sensitivity, both of which are affected by olanzapine [26]. Present review from three clinical trials showed that the weight gain of metformin group was less than the placebo group (*z* = −2.464, *P* = 0.014). Adverse effects were tolerable, in accordance with previous researches about metformin, whereas two subjects were withdrawn from the trials for lack of response. Though it was rare, data regarding the metformin indicated potential fatal side effect of lactic acidosis, particularly in elderly and those with compromised renal function, and its new-found association with the accumulation of beta-amyloid, a factor in the pathogenesis of Alzheimer's disease, alter the risk-benefit ratio in the elderly [4].

Aripiprazole is a partial agonist at D_2_ dopamine and 5-HT_1A_ serotonin receptors and an antagonist at 5-HT_2A_ and 5-HT_2C_ serotonin receptors. In long-term studies in schizophrenia, aripiprazole treatment has not been associated with a mean increase in body weight from baseline; in some studies, small decreases in mean body weight have been observed [27, 28]. Karunakaran et al. [29] reported an average weight loss of 5.1 kg among patients who had aripiprazole added to decreased doses of clozapine over 34 weeks. Henderson et al. [30] concluded that the addition of aripiprazole to a stable dose of olanzapine was well tolerated and resulted in significant decreases in weight and BMI compared with placebo. There was no significant change in total PANSS or another clinical psychopathology. Present review of aripiprazole (*n* = 207) was in-line with above trials in terms of weight reduction in which −2.5 kg of mean change from baseline was shown in the aripiprazole group. There were no significant differences in PANSS score changes between groups but Clinical Global Impression Improvement and Investigator's Assessment Questionnaire scores favored aripiprazole over placebo [30]. On the other hand, the adverse effects were worse than previous trials: ten patients complained about severe adverse effects and three of them developed moderate sinus tachycardia, severe psychotic disorder, and severe auditory hallucinations [28, 29]. Thus, combining aripiprazole and clozapine may be in significant weight reduction to patients suboptimally treated with clozapine, while this has to be balanced against the higher likelihood of the aripiprazole and clozapine combination to induce side effects such as nausea, anxiety, and akathisia [8].

Topiramate has been associated with weight loss as a side effect by reduced appetite related to the mechanism of potentiating GABAergic transmission and antagonism of AMPA glutamate receptors; its precise mechanism remains under investigation [31]. Several researchers have described that topiramate adjunctive treatments appeared to be scarcely effective for reducing weight and clinical symptoms and even induce exacerbations of symptoms [6, 32]. Other studies reported that topiramate adjunctive treatments led to improvement in psychiatric symptoms, and decrease in body weight, which were generally well tolerated [33]. Present study showed that topiramate adjunctive treatments were effective on weight reduction (*z* = −2.916, *P* = 0.004) as well as improvement of psychiatric symptoms evaluated by PANSS (*P* ≤ 0.001). Adverse effects were mild to moderate in severity with no serious adverse effects evidenced by no treatment withdrawal due to these side effects [9, 10]. Topiramate may prevent olanzapine induced weight gain and adverse metabolic effects [9], and its clinical response (more than 20% reduction in PANSS) is significantly higher than those of control groups (50% versus 12.5%) [10]. Therefore, topiramate adjunctive treatment can be effective in reduction of body weight induced by atypical antipsychotics as well as controlling schizophrenic symptoms [10].

Limitations of this study include that small number of trials have been selected as relevant trials among 131 trials, which may prohibit direct application or generalization of the results. In addition, there is still controversy regarding weight gain and metabolic side effects across available antipsychotic drugs showing distinction between typical and atypical drugs. Thus, in this study, we have focused on the issue related with olanzapine and clozapine, which have evidences of side effects concerning weight gain and metabolic syndrome, which needs conscious deliberation on discourse concerning results of this study.

## 5. Conclusion

Adjunctive treatments of metformin, sibutramine, topiramate, and aripiprazole are significantly effective to reduce weight for patients receiving atypical antipsychotics. While it presented larger weight reduction, sibutramine is not a choice of option, which was withdrawn from the US market for critical cardiovascular adverse events. Although metformin outperformed other agents that have been studied against placebo, the current evidence is too limited to support its regular clinical use as an adjunctive medication and it needs additional proofs of safety regarding Alzheimer's disease. Though having several reports related to exacerbation of psychiatric symptoms, topiramate and aripiprazole are more efficacious than other medications in regard to weight reduction and less burden of critical adverse effects as well as being beneficial for clinical improvement. Results of this study provide opportunity to consider adjunctive treatments added to atypical antipsychotics for weight control and/or metabolic syndrome in patients with schizophrenia.

## Figures and Tables

**Table 1:** Characteristics of studies for systematic review and meta-analysis.

Number	First author	Jadad score	Concurrent therapy	Augmentation (mg/day, daily max. dose)	Duration (weeks)	*n* (drop)	Primary outcome	*P*	Secondary outcome	*P*
1	Fleischhacker (2010) [8]	4	Clozapine	Aripiprazole (15) placebo	16	108 (0) 99 (0)	BWT	<0.001	PANSS	0.37

2	Narula (2010) [9]	3	Olanzapine	Topiramate (100) placebo	12	33 (3) 34 (2)	BMI	0.004	PANSS	0.00

3	Afshar (2009) [10]	4	Clozapine	Topiramate (300) placebo	8	16 (0) 16 (0)	BMI	≤0.05	PANSS	<0.001

4	Baptista (2008) [11]	4	Olanzapine	Metformin (1700) + sibutramine (20) placebo	12	13 (0) 15 (0)	BWT	0.19	BPRS	0.15

5	Wu (2008) [12]	5	Olanzapine	Metformin (750) placebo	12	20 (2) 20 (1)	BWT	<0.02	SAPS	0.52

6	Baptista (2007) [13]	4	Olanzapine	Metformin (2250) placebo	12	40 (4) 40 (4)	BWT	0.09	BPRS	ns

7	Henderson (2007) [14]	5	Clozapine	Sibutramine (15) placebo	12	10 (0) 11 (3)	BWT	0.31	PANSS	0.96

8	Poyurovsky (2007) [15]	5	Olanzapine	Reboxetine (4) placebo	6	31 (9) 28 (9)	BWT	0.013	SAPS	0.96

9	Baptista (2006) [16]	4	Olanzapine	Metformin (1700) placebo	14	20 (1) 20 (2)	BWT	0.4	BPRS	ns

10	Henderson (2005) [7]	4	Olanzapine	Sibutramine (15) placebo	12	19 (3) 18 (3)	BWT	0.009	PANSS	ns

11	Poyurovsky (2004) [17]	3	Olanzapine	Famotidine (40) placebo	6	7 (0) 7 (0)	BWT	0.91	SAPS	0.54

12	Cavazzoni (2003) [18]	2	Olanzapine	Nizatidine (300) placebo	16	57 (0) 60 (0)	BWT	0.36	BPRS	0.63

13	Poyurovsky (2003) [19]	3	Olanzapine	Reboxetine (4) placebo	6	13 (3) 13 (3)	BWT	0.03	SAPS	0.83

14	Poyurovsky (2002) [20]	3	Olanzapine	Fluoxetine (20) placebo	8	15 (4) 15 (2)	BWT	0.44	SAPS	0.83

**(a):** 

Number	First author	Concurrent therapy	Adjunctive agent (mg/day, daily max. dose)	Duration (weeks)	*n* (drop)	Primary outcome				Secondary outcome			
						Weight reduction	Mean outcome	Mean difference	*P*	Psychiatric evaluation	Mean baseline	Mean change	*P*
2	Narula (2010) [9]	Olanzapine	Topiramate (100) placebo	12	33 (3) 34 (2)	BMI (kg/m^2^)	20.1 22.6	−2.5	0.004	PANSS	102.9 103.8	−71.7 −70.5	0.001

3	Afshar (2009) [10]	Clozapine	Topiramate (300) placebo	8	16 (0) 16 (0)	BMI (kg/m^2^)	23.2 25.4	−2.19	≤0.05	PANSS	96.9 101.9	−20 −1.3	<0.001

**(b):** 

Number	First author	Statistics for each study					Difference in means and 95% CI	Relative weight
		Difference in means	Standard error	Variance	*Z* value	*P* value		
2 3	Narula (2010) [9] Afshar (2009) [10] Total	−2.500 −2.190 −**2.405**	0.990 1.491 **0.825**	0.980 2.223 **0.680 **	−2.525 −1.469 −**2.916**	0.012 0.142 **0.004**	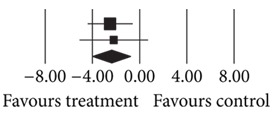	69.41 30.59 ** 100.00**

Heterogeneity: *Q* = 0.03; df = 1; *P* = 0.86; *I* ^2^ = 0.00.

**(a):** 

Number	First author	Concurrent therapy	Adjunctive agent (mg/day, daily max. dose)	Duration (weeks)	*n* (drop)	Primary outcome				Secondary outcome			
						Weight reduction	Mean baseline	Mean change	*P*	Psychiatric evaluation	Mean baseline	Mean change	*P*
7	Henderson (2007) [14]	Clozapine	Sibutramine (15) placebo	12	10 (0) 34 (2)	BWT (kg)	104.8 102.1	−1.9 −0.5	0.310	PANSS	63 62	5.3 −4.4	0.96

10	Henderson (2005) [7]	Olanzapine	Sibutramine (15) placebo	12	19 (3) 18 (3)	BWT (kg)	102.7 109	−6.8 3.9	0.009	PANSS	54.9 57.4	−1.9 −5.5	ns

**(b):** 

Number	First author	Statistics for each study					Difference in means and 95% CI	Relative weight
		Difference in means	Standard error	Variance	*Z* value	*P* value		
7 10	Henderson (2007) [14] Henderson (2005) [7] Total	−1.400 −2.900 **−2.342**	1.335 1.027 **0.814**	1.783 1.055 **0.663**	−1.048 −2.823 **−0.747**	0.294 0.005 **0.004**	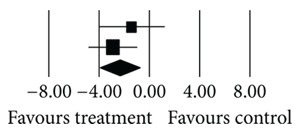	37.18 62.82 **100.00**

Heterogeneity: *Q* = 0.79; df = 1; *P* = 0.37; *I* ^2^ = 0.00.

**(a):** 

Number	First author	Concurrent therapy	Adjunctive agent (mg/day, daily max. dose)	Duration (weeks)	*n* (drop)	Primary outcome				Secondary outcome			
						Weight reduction	Mean baseline	Mean change	*P*	Psychiatric evaluation	Mean baseline	Mean change	*P*
5	Wu (2008) [12]	Olanzapine	Metformin (750) placebo	12	20 (0) 20 (1)	BWT (kg)	55.7 56.5	1.9 6.9	<0.02	SAPS	8.5 7.9	−6.3 −5.8	0.52

6	Baptista (2007) [13]	Olanzapine	Metformin (2250) placebo	12	40 (4) 40 (4)	BWT (kg)	66.2 65.6	−1.4 −0.2	0.090	BPRS	15.2 14.7	1.1 −0.2	ns

9	Baptista (2006) [16]	Olanzapine	Metformin (1700) placebo	14	20 (1) 20 (2)	BWT (kg)	58.3 59.4	5.5 6.5	0.4	BPRS	18.3 17.4	−6.6 −3.9	ns

**(b):** 

Number	First author	Statistics for each study					Difference in means and 95% CI	Relative weight
		Difference in means	Standard error	Variance	*Z* value	*P* value		
5 6 9	Wu (2008) [12] Baptista (2007) [13] Baptista (2006) [16] Total	−5.000 −1.200 −0.800 **−1.331**	2.051 0.698 0.939 **0.540**	4.207 0.487 0.882 **0.292**	−2.438 −1.719 −0.852 **−2.464**	0.015 0.086 0.394 **0.014**	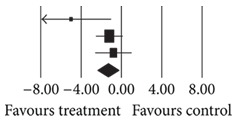	6.94 59.94 33.12 **100.00**

Heterogeneity: *Q* = 3.55; df = 2; *P* = 0.17; *I* ^2^ = 43.74.

**(a):** 

Number	First author	Concurrent therapy	Adjunctive agent (mg/day, daily max. dose)	Duration (weeks)	*n* (drop)	Primary outcome				Secondary outcome			
						Weight reduction	Mean baseline	Mean change	*P*	Psychiatric evaluation	Mean baseline	Mean change	*P*
8	Poyurovsky (2007) [15]	Olanzapine	Reboxetine (4) placebo	6	31 (9) 28 (9)	BWT (kg)	67.1 68.4	3.3 4.9	0.013	SAPS	6.4 5.8	−3.2 −3.1	0.96

13	Poyurovsky (2003) [19]	Olanzapine	Reboxetine (4) placebo	6	13 (3) 13 (3)	BWT (kg)	65.8 59.8	2.4 5.4	0.03	SAPS	23.2 31.7	−18.4 −19.9	0.83

**(b):** 

Number	First author	Statistics for each study					Difference in means and 95% CI	Relative weight
		Difference in means	Standard error	Variance	*Z* value	*P* value		
8 13	Poyurovsky (2007) [15] Poyurovsky (2003) [19] Total	−1.600 −3.000 **−1.862**	0.624 1.300 **0.563**	0.389 1.691 **0.316**	−2.564 −2.307 **−3.310**	0.010 0.021 **0.001**	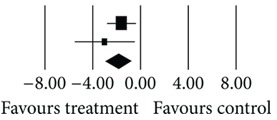	81.29 18.71 **100.00**

Heterogeneity: *Q* = 0.94; df = 1; *P* = 0.33; *I* ^2^ = 0.00.
